# Complete Genome Sequence of a New Strain of Tanay Virus, Isolate YN15_103_01, from Yunnan, China

**DOI:** 10.1128/genomeA.00058-18

**Published:** 2018-02-22

**Authors:** Lu Zhao, Han Xia, Yujuan Wang, Atoni Evans, Yunzhi Zhang, Zhiming Yuan

**Affiliations:** a Key Laboratory of Special Pathogens and Biosafety, Wuhan Institute of Virology, Chinese Academy of Sciences, Wuhan, Hubei, China; b Southern University of Science and Technology, Shenzhen, Guangzhou, China; c Yunnan Provincial Institute of Endemic Disease Control and Prevention, Dali, Yunnan, China

## Abstract

We report here the complete genome sequence of a new strain of Tanay virus, isolate YN15_103_01, which was isolated from Anopheles sinensis mosquitoes caught in China. Sequence analysis showed that it shares a 79% nucleotide sequence identity with the only other previously reported strains of Tanay virus, which were isolated from the Philippines.

## GENOME ANNOUNCEMENT

Tanay virus (TANAV) is a positive-sense single-stranded RNA virus with a poly(A) tail and is a member of the group Nege-CAB, which has not been classified yet. Our findings follow the discovery of TANAV from the Philippines in 2013 (1).

We report here the full-genome sequence of a new strain of Tanay virus, isolate YN15_103_01, which was isolated for the very first time in China from Anopheles sinensis. To determine the full-genome sequence of the isolate, total RNA was extracted using an RNeasy minikit (Qiagen, Germany), and strand-specific libraries were prepared using the TruSeq stranded total RNA sample preparation kit (Illumina, USA) following the manufacturer’s instructions. Sequencing was performed at Shanghai Biotechnology Corporation using the Illumina HiSeq 2500 (Illumina, USA) platform. Generated sequence reads were *de novo* assembled using Trinity on the Galaxy platform (http://mississippi.snv.jussieu.fr), and a 9,580-nt-long contig was generated. To validate the results of the sequenced reads, 6 pairs of PCR primers and 36 pairs of sequencing primers were designed based on the sequence information of the contig. The PCR products were puriﬁed and cloned into the pMD18-T vector (TaKaRa) and sent for sequencing. To acquire the terminal sequences, both 3′ and 5′ rapid amplification of cDNA ends (RACE) procedures were conducted using the SMARTer RACE 5′/3′ kit user manual (TaKaRa, Japan). Putative opening reading frame (ORF) organization was predicted by the Gene Finding in Viral Genomes function in the Softberry software program.

The complete genome sequence of TANAV isolate YN15_103_01 is 9,587 nucleotides (nt) in size, with 79% nt identity to the previously reported isolates (GenBank accession numbers KF425261, KF425262, KF425263, and KF425264). There are three putative opening reading frames, as follows: ORF1 comprises 2,215 amino acids (aa) ranging from nt positions 78 to 6,725; ORF2 comprises 594 aa ranging from nt positions 6,749 to 8,533; and ORF3 comprises 215 aa ranging from nt positions 8,663 to 9,310.

### Accession number(s).

The nucleotide sequence of TANAV isolate YN15_103_01 identified in this study has been deposited in GenBank under accession number MG673930.
